# Histone Deacetylase Inhibitor Combined with Rosiglitazone Improves Cognitive Function Via Microglial Polarization and Increased Mature/Pro-BDNF in Alzheimer’s Disease

**DOI:** 10.34172/apb.025.46129

**Published:** 2025-12-13

**Authors:** Melina Rafiey, Rahim Nosrati, Arash Pourgholaminejad, Saba Ahangaran, Parvin Babaei

**Affiliations:** ^1^Neuroscience Research Center, Trauma Institute, School of Medicine, Guilan University of Medical Sciences, Rasht, Iran; ^2^Cellular and Molecular Research Center, School of Medicine, Guilan University of Medical Sciences, Rasht, Iran; ^3^Department of Medical Immunology, School of Medicine, Guilan University of Medical Sciences, Rasht, Iran; ^4^Department of Physiology, School of Medicine, Guilan University of Medical Sciences, Rasht, Iran

**Keywords:** Alzheimer’s disease, Microglial polarization, PPAR γ, Rosiglitazone, HDAC3i, MS-275

## Abstract

**Introduction:**

Alzheimer’s disease (AD) is characterized by diminished brain metabolism, cognitive impairments, neural loss, astrogliosis, and microgliosis. We hypothesized that co-administration of a peroxisome proliferator-activated receptor gamma (PPARγ) agonist and a histone deacetylase (HDAC) inhibitor would enhance cognitive function in an AD model of rats.

**Methods:**

Forty adult male Wistar rats were randomly assigned into five groups (n=8 per group): (1) Control group receiving saline, (2) AD model group (induced by i.c.v injection of Streptozocin), (3) AD+Rosiglitazone (ROSI) (4) AD+MS-275, and (5) AD+combined ROSI and MS-275 group. Cognitive functions were evaluated using the passive avoidance test and the Morris water maze (MWM). Microglial polarization was assessed by flow cytometry, and protein expression was analyzed by western blotting.

**Results:**

Data analyzed by one-way ANOVA and post hoc Tukey for (MWM) showed a significant decrease in latency to the target quadrant both in working and reference memories, and a significant increase in total time spent (TTS) in the target quadrant for reference memory in the group of STZ+ROSI+MS-275 (*P*<0.000). Kruskal-Wallis H test revealed a significant increase in the M2/M1 ratio for ROSI+MS-275+STZ group compared with the STZ+Saline group (*P*=0.001). Also an increased mature brain-derived neurotrophic factor (BDNF)/pro-BDNF ratio was found in treated groups compared with STZ+saline (*P*<0.001).

**Conclusion:**

These findings suggest that co-administration of Rosiglitazone and MS-275 improves cognitive function in AD rats, potentially through shifting microglial polarization from the M1 to the M2 phenotype and enhancing synaptic strength via an increased mature BDNF/pro-BDNF ratio.

## Introduction

 Alzheimer’s disease (AD) is a neurodegenerative condition with a complex etiology and unique neuropathological characteristics. AD represents 60-70% of all dementia cases in those aged 65 and older.^1^ Despite remarkable achievements on the pathophysiological hallmarks of AD,^2^ the initiating factor has not been clarified yet. Among various hypotheses, neuroinflammation mediated by brain glial cells and brain glucose metabolism disturbance has great importance. Glucose is the sole fuel for the brain,^3^ and is reduced at the early phase of cognitive decline in AD.^4^ Therefore, astrocytes and microglia shift their metabolism to anaerobic glycolysis, which causes a reduction in adenosine triphosphate (ATP),^5^ that is consequently followed by an increase in neuroinflammation, synaptic dysfunction, and cognitive decline.^6^

 Microglia demonstrate plasticity with at least two distinct phenotypes: M1 is pro-inflammatory, which releases inflammatory cytokines, leading to the distribution of amyloid β and elevation in proinflammatory mediators;^7^ M2 is anti-inflammatory, it releases neurotrophic^8^ and anti-inflammatory factors,^9^ including BDNF.^10^ M1 microglia exhibit common cell surface markers such as CD86, CD68, and CD80,^11^ while M2 anti-inflammatory microglia exhibit CD163 and CD206.^12^

 On the other hand, peroxisome proliferator-activated receptors (PPARs) agonists are potent insulin sensitizers, which play a neuroprotective role^13^ through target genes,^14^ particularly NF-κB.^15^ Rosiglitazone, as a selective ligand for PPARγ, removes Aβ from the surrounding environment^16^ and also inhibits microglia activation in response to lipopolysaccharide.^17^ However, the clinical utility of this agent is constrained by side effects, including fluid retention, increased weight, bone loss, and low penetrability through the blood-brain barrier (BBB).^18^ To mitigate these limitations, we explored the combined use of rosiglitazone with a histone deacetylase inhibitor (HDACi) to enhance neuroprotective efficacy. HDACi can remodel chromatin and regulate gene transcription, particularly genes implicated in memory retention and inflammation.^19^

 To establish the experimental model, we used streptozocin (STZ) to induce sporadic AD through elevating oxidative stress, promoting hyperphosphorylated Tau^20^ and increasing insulin resistance.^21^ Spatial memory and aversive learning were assessed by MWM and the passive avoidance learning test, respectively. Microglial polarization and BDNF level in the hippocampus were measured using flow cytometry and western blot analysis.

## Materials & Methods

###  Animal Grouping

 Forty adult male Wistar rats (220–270 g) were obtained from the College of Pharmacy and kept four per cage. The animals were maintained under controlled conditions (22°C; 12 h light/dark cycle, lights on at 07:00 h) with free access to standard food and water. All of the protocols were reviewed and accepted by the Ethics Committee of Guilan University of Medical Sciences (approval code: IR.GUMS.AEC.1401.009) in accordance with the European Communities Council Directive (86/609/EEC).

**Figure 1:**
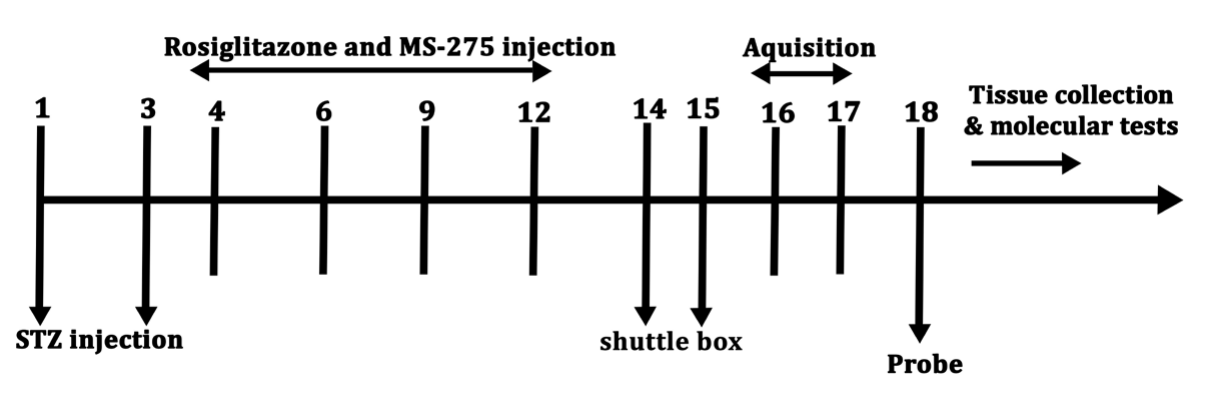
Diagram representation of the experimental timetable

 The rats were allocated into five groups in random order (n = 8 per group): Control group receiving saline (STZ vehicle), AD model group (STZ), AD rats receiving either Rosiglitazone (ROSI) or MS-275, and finally AD rats with co-treatment of both Rosiglitazone and MS-275 (Figure 1).

###  Stereotaxic Surgery And Injections

 A mixture of xylazine (10 mg/kg) and ketamine (65 mg/kg) was used to anesthetize the rats, which were afterwards positioned in a stereotaxic device. The guide cannula, a 23-gauge stainless steel, was then implanted bilaterally into the ventricles via a cranial opening at the following coordinates of stereotaxic: AP = 0.8 mm, DV = 3.8 mm, and MD = 1.7 mm.^22^

###  Drug Preparation and Microinfusing

 Streptozocin (Sigma-Aldrich, St. Louis, MO, USA) (3 mg/kg) was dissolved in saline, and then administered by intracerebroventricular (i.c.v) injection with a Hamilton micro syringe into the guide cannulas within 5 minutes at one-day intervals.^23^

 MS-275 (Cat No: 13284 - Cayman, USA) was mixed with saline and 1% DMSO,^24^ and injected (i.c.v).^19^ Rosiglitazone (Cat No: 122320-73-4 Cayman Chemical, USA) was watered down in a 70% isotonic saline and 5% DMSO solution, and then injected (i.c.v) with a dosage of 100 μg/kg in 5 μL.^25^

###  Behavioral Evaluations

####  Morris Water Maze

 Cognitive function was measured using the Morris Water Maze (MWM) test, as described in our previous work.^26^ The setup was a circular pool (diameter: 148 cm, height: 60 cm, depth: 25 cm) filled with room-temperature water, and a circular escape platform (diameter: 10 cm) placed 2 cm underneath the surface of water. A digital high-resolution camera was positioned above the pool to record animal performances, which were afterwards analyzed using the EthoVision tracking system (Noldus, Netherlands). The pool was virtually divided into four quadrants (southwest, southeast, northwest, and northeast). During the working memory training phase, the hidden platform was located in the middle of the southwest section. Each rat completed four blocks of trials (16 in total), with a 20-minute rest between blocks. The average latency to locate the platform at different start points was calculated. Long-term memory was assessed by comparing the TTS in the target quadrant versus the other three quadrants, as well as the latency to find the platform within a 90-second trial period.

####  Step-Through Passive Avoidance Learning

 Step-through Passive Avoidance Learning test was conducted using a single-trial protocol.^21^ The apparatus consisted of two adjoining compartments, an illuminated and a dark one, divided by a guillotine gate. Each animal was positioned alone in the lighted compartment with a closed door and allowed to explore for 20 seconds. The animal was then permitted to enter the dark section, where a brief foot shock (0.5 mA) was delivered through the grid floor. After escaping back to the light chamber, the rat was returned to its home cage. Twenty-four hours later, the test was performed again, but without a shock, and both the latency to re-enter the dark compartment and the TTS there during a 300-second trial were recorded.

###  Tissue Collection and Preservation

 The animals were euthanized by exposure to CO2 in a closed desiccator and then sacrificed by decapitation. The hippocampus was removed on an ice surface and then stored at -80°C for subsequent use in Western blot and flow cytometry.

####  Flow Cytometry and Cell Staining Approach

 To determine the macrophage polarization, the extracted cells from the hippocampus tissue were analyzed by a flow cytometry approach. Briefly, the tissue was cleaned and cut into small pieces, and then was fixed with paraformaldehyde for 20 minutes. Afterward, the tissue was washed with PBS following the removal of supernatant to generate a single-cell suspension. To identify the microglia subtypes (M1 and M2), appropriate primary and conjugated-secondary antibodies were used in the staining protocol according to the manufacturer’s protocol. To detect CD163 (M2 marker), primary unconjugated rabbit anti-mouse CD163 (Biorbyt, UK) with the following PE-conjugated goat anti-rabbit IgG/PE (SouthernBiotech, US) were used. Then, to detect CD86 (M1 marker), primary unconjugated mouse anti-mouse CD86 (Biorbyt, UK) with the following FITC-conjugated rabbit anti-mouse IgG/FITC (Biorbyt, UK) were used according to the manufacturer’s protocol. All flowcytometry tubes were matched with appropriate isotype-control antibodies. Furthermore, the data were collected by BD FACSCalibur Flow cytometer and analyzed with FlowJo software (FlowJo LLC, Ashland, OR, USA) version 7.6.1.

####  Western Blot

 Protein lysates were prepared in 500 µL of ice-cold RIPA buffer (Bio-Rad, USA) supplemented with 1 µL of protease inhibitor. Total protein was taken out, and sample concentrations were quantified using the Bradford assay (Bio-Rad, USA). Equal amounts of protein were mixed with loading buffer and denatured at 100 °C for 5 minutes, followed by separation on 10% SDS-PAGE gels (1.5 h at 110 V). Proteins were transferred onto a membrane of polyvinylidene fluoride (PVDF) via a wet transfer system. Membranes were blocked and incubated with primary antibodies for BDNF (sc-65514, Santa Cruz Biotechnology) and β-actin (sc-47778, Santa Cruz Biotechnology) at a dilution of 1:1000 for 16 h at 4 °C with agitation. After washing with TBST, they were incubated with secondary antibodies, including HRP-conjugated anti-rabbit IgG (sc-2357, Santa Cruz Biotechnology) and mouse IgGκ binding protein (sc-516102, Santa Cruz Biotechnology), with a dilution of 1:2000 for 2 h at 25°C. After additional washes, signals were visualized using enhanced chemiluminescence (ECL) reagents, and band intensities were determined via X-ray film exposure.^19^ The Western blot bands were quantified using densitometry analysis with Image J software, and the intensity values were normalized to β-actin as a control protein.

###  Statistical Analyses

 Shapiro and Kolmogorov-Smirnov tests were used to assess the normality of data in SPSS and showed normal distribution (*P* > 0.05). Group comparisons were done by means of either one-way or two-way analysis of variance (ANOVA), with Tukey and LSD post hoc tests afterwards. Flow cytometry data were studied by the Kruskal-Wallis H test, with post-hoc test of Bonferroni-adjusted pairwise comparison, as the data did not meet the normality requirements for the one-way ANOVA test. Test data are expressed as the mean ± SEM (standard error of the mean). Significance was defined as *P* < 0.05.

## Results

###  Behavioral Evaluations in MWM

####  Co-Treatment of MS-275 and Rosiglitazone Improves Working Memory in MWM

**Figure 2:**
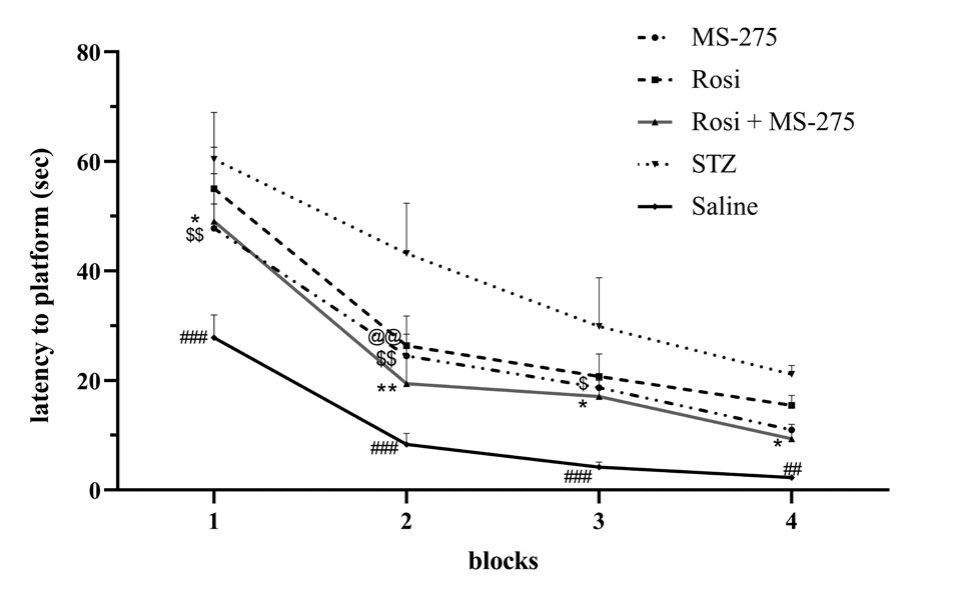
Two-way ANOVA followed by LSD’s *post-hoc* tests was used for statistical analysis, and data are presented as mean ± SEM (n = 8). *P *<*0.05, **P *<*0.01, ***P *<*0.001 compared with STZ group

 Statistical analysis by Two-way ANOVA (groups × blocks) revealed a significant blocks effect on escape latency [F (3,156) = 79.85, *P* < 0.001] and also on group effect [F (4,35) = 26.97, *P* < 0.001, Figure 2]. Between groups interaction was not significant in the MWM arena [F (12,156) = 0.781, *P* = 0.670].

 LSD’s post-hoctests exposed a substantial decrease (40%) in escape latency for STZ + ROSI + MS-275 group [F (4,35) = 26.975, *P* = 0.001], a 33.5% decrease for STZ + MS-275 [F (4,35) = 26.975, *P* = 0.001] group, and 23% for the group of STZ + ROSI [F (4,35) = 26.975, *P* = 0.001] in comparison with the STZ + saline group. There was a significant difference between co-treatment and STZ + ROSI [F (4,35) = 26.975, *P* = 0.031]. LSD’s post-hoc tests revealed a noteworthy decrease in latency of control, STZ + ROSI + MS-275, and STZ + MS-275 groups in blocks 1- 4 (*P* < 0.05), while the STZ + ROSI group only in block 2 (*P* < 0.05) compared with the STZ + Saline (Figure 2).

####  Effect of Chronic Hippocampal Injection of Rosiglitazone and MS-275 on Spatial Memory Recovery in the Probe Test

**Figure 3:**
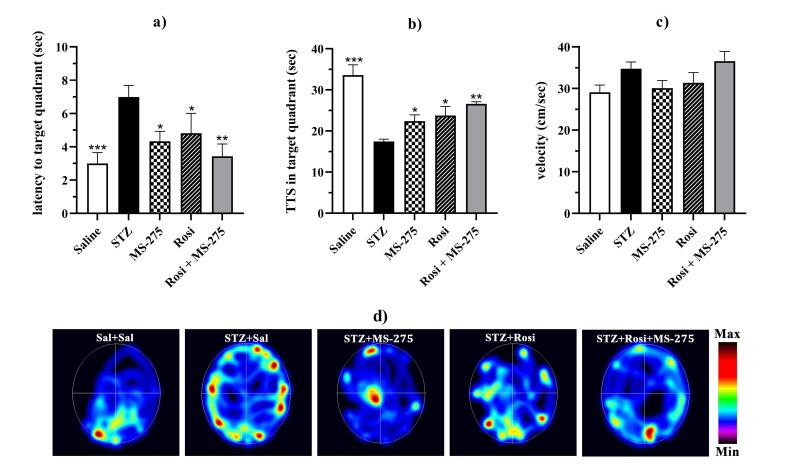
Probe test. a) latency to target quadrant, b) total time spent in the target quadrant, c) swimming speed, d) heat maps showing trailing of rats in the retrieval test. One-way ANOVA followed by LSD’s *post-hoc* tests was used for statistical analysis, and data are presented as mean ± SEM (n = 8). *P *<*0.05, **P *<*0.01, ***P *<*0.001 compared with STZ + Saline group. Increasing color intensity signifies increased time spent in the acquisition procedure

 Data studied by one-way ANOVA along with LSD’s post-hoc tests showed significant between-group alterations in latency to target quadrant [F (4,35) = 4.948, *P* *<*0.001] and TTS in the target quadrant during probe test [F (4,35) = 12.289, *P* = 0.001]. Moreover, LSD’s *post-hoc *tests comparisons showed that latency was decreased in STZ + ROSI + MS-275 [F (4,35) = 4.948, *P* = 0.001], STZ + MS-275 [F (4,35) = 4.948, *P* = 0.01] and STZ + ROSI [F (4,35) = 4.948, *P* = 0.034] compared to STZ + Saline, Figure 3a. Longer TTS was achieved for STZ + ROSI + MS-275 [F (4,35) = 12.289, *P* = 0.001], STZ + ROSI [F (4,35) = 12.289, *P* = 0.012], and STZ + MS-275 [F (4,35) = 12.289, *P* = 0.047] in comparison with STZ group, Figure 3b. Although co-treatment group showed better performance, it had no significant difference with monotherapy groups.

 In the assessment of the swimming speed of groups, no significant difference was found [F (4,35) = 2.409, *P* = 0.068, Figure 3c].

 Collectively, these results show the additive effects of MS-275 and Rosi on reference memory.

###  Step Through Passive Avoidance Learning

####  Effect of Co-Treatment of Rosiglitazone and MS-275 on Passive Avoidance Memory Preservation in STZ-Induced Rats

**Figure 4:**
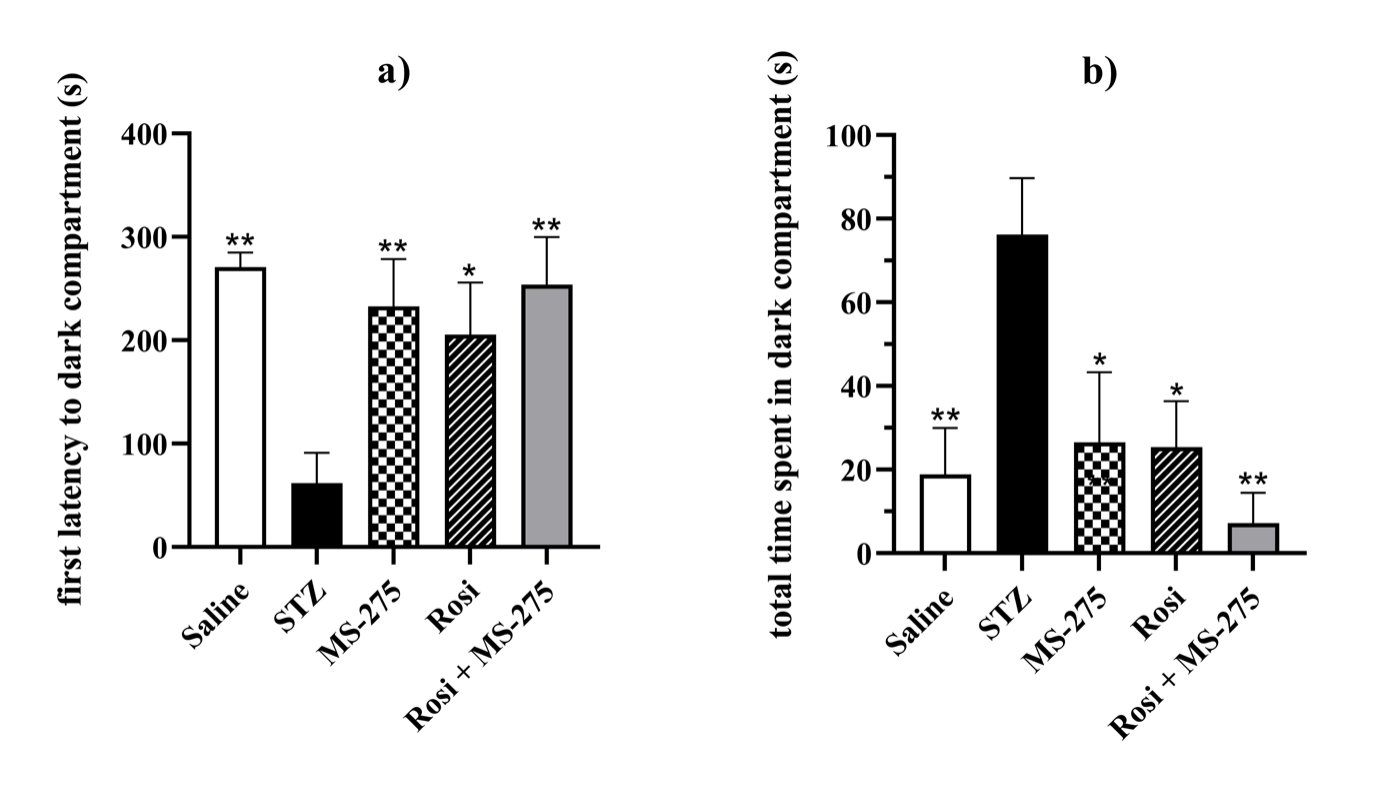
Passive avoidance memory analysis. a) First latency to dark compartment, b) total time spent in the dark compartment. One-way ANOVA followed by LSD’s *post-hoc *tests was used, and data are presented as mean ± SEM (n = 8) compared with STZ + Saline group, *P *<*0.05, **P *<*0.01, ***P *<*0.001

 One-way ANOVA assessment displayed substantial between-group differences in the first latency to the dark chamber [F (4,35) = 4.229, *P* = 0.01]. Comparison with LSD’s post-hoctest displayed a significant increase in latency to the dark room for STZ + ROSI [*P* = 0.023], STZ + MS-275 [*P* = 0.006], and STZ + ROSI + MS-275 [*P* = 0.002] compared with STZ group. No significant difference was found in the first latency to the dark compartment among these groups (*P* > 0.05), Figure 4a.

 In addition, one-way ANOVA and LSD’s post-hoc test showed a noteworthy decrease in TTS in the dark room for STZ + ROSI [*P* = 0.012], STZ + MS-275 [*P* = 0.010], and STZ + ROSI + MS-275 [*P* = 0.001] compared with the group of STZ + saline. Similar to MWM, co-treatment had achieved a slightly and insignificant better performance, Figure 4b.

###  Flow Cytometry

####  Co-Administration of Rosiglitazone and MS-275 Results in Polarization of M1 Microglia Towards M2 Phenotype

**Figure 5:**
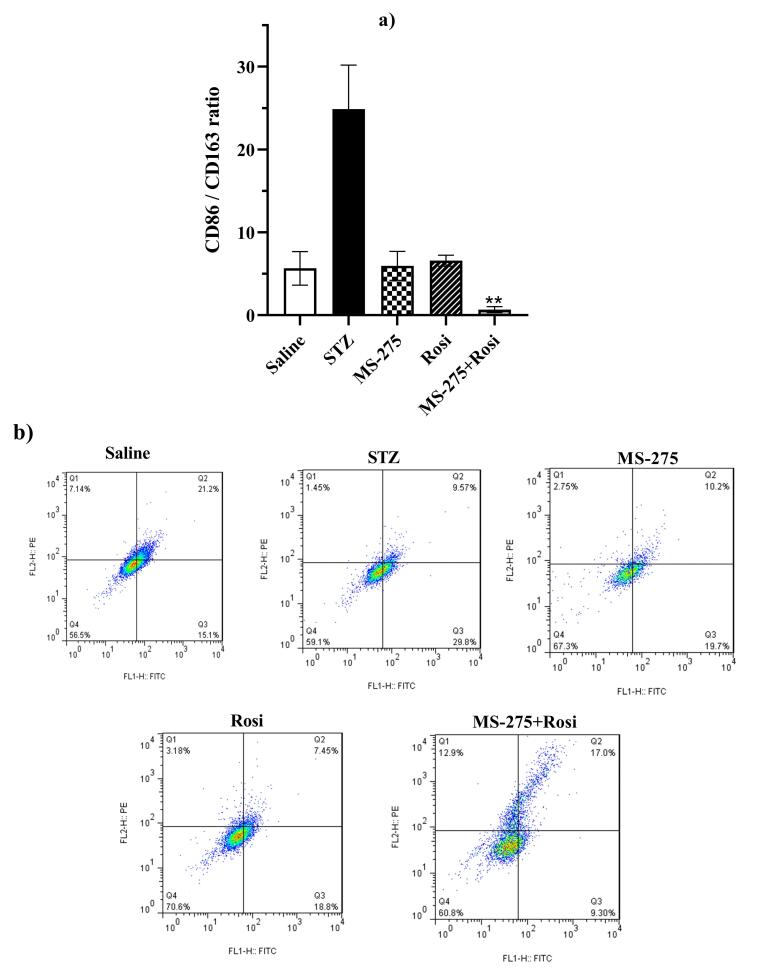
CD86 and CD163 represent M1 and M2 phenotypes, respectively. a) M1/M2 (CD86/CD163) ratio, and b) representative quadrant flow cytometry dot-plots. Kruskal-Wallis test with post-hoc test using Bonferroni adjusted pairwise comparison was used, and data are shown as mean ± SEM (n = 3) compared with STZ + Saline group, *P *<*0.05, **P *<*0.01, ***P *<*0.001

 Assessment of polarization of M1 to M2 (M1/M2 ratio) was done by comparing CD86/CD163, CD86 as a marker for M1, and CD163 as a marker for M2 (Figure 5a, 5b). Kruskal-Wallis H test exhibited a momentous difference between groups of rats [H (4) = 10.448, *P* = 0.034]. Pairwise comparison revealed a significant decrease in the M1/M2 ratio for ROSI + MS-275 + STZ [*P* = 0.001] in relation to STZ + Saline group; the same result was achieved with Bonferroni-adjusted post hoc correction P value [*P* = 0.013, Figure 5a]. Also, no significance was seen between ROSI + STZ [*P* = 0.116, Bonferroni-adjusted *P* = 1.000] and MS-275 + STZ [*P* = 0.097, Bonferroni-adjusted *P* = 0.971] compared with the co-treatment group.

###  Western Blot

**Figure 6:**
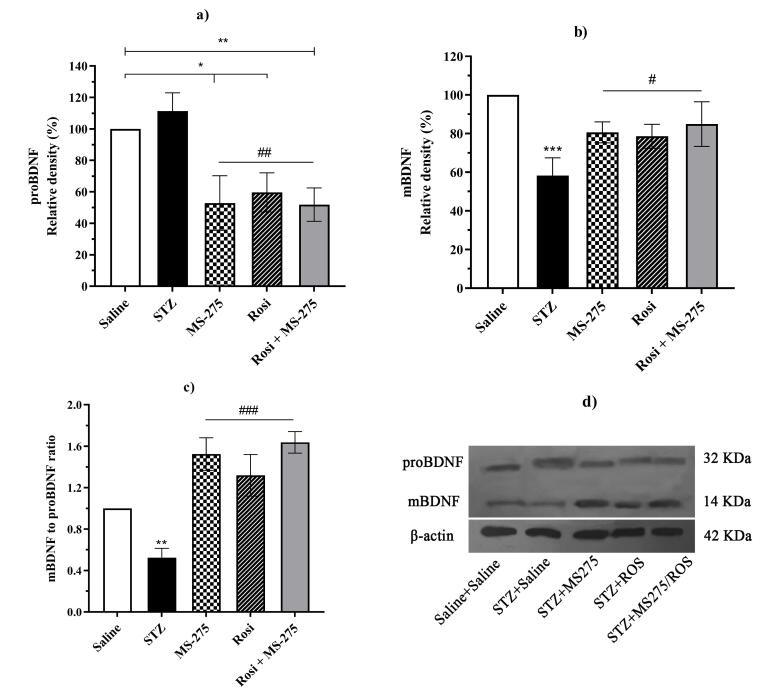
Western blot analysis of a) pro-BDNF expression, b) m-BDNF expression in different groups normalized with β-actin as a protein loading, c) m-BDNF to pro-BDNF ratio, and d) specific bands and quantification of pro-BDNF, m-BDNF, and β-actin. One-way ANOVA followed by Tuckey’s *post-hoc* tests was used for statistical analysis, and data are presented as mean ± SD (n = 3). *P *<*0.05, **P *<*0.01, ***P *<*0.001 compared with Saline group and #*P* < 0.05, ##*P* < 0.01, ###*P* < 0.001 compared with STZ group. d) Western blot bands density of pro and mature BDNF in the hippocampus

 Quantitation analysis of proand mature BDNF protein bands density from western has been shown in Figure 6a, b, c, d. Assessment by one-way ANOVA along with Tukey’s post-hoc test revealed a significant difference between groups in pro-BDNF [F (4,10) = 17.22, *P* < 0.001], m-BDNF [F (4,10) = 11.86, *P* < 0.001], and m-BDNF to pro-BDNF ratio [F (4,10) = 36.10, *P* < 0.001]. Pro-BDNF level was significantly reduced in STZ + ROSI + MS-275 [F (4,10) = 17.22, *P* < 0.001], STZ + MS-275 [F (4,10) = 17.22, *P* < 0.001], and STZ + ROSI [F (4,10) = 17.22, *P* = 0.002] in association with STZ group, Figure 6a. Additionally, no significant difference was found in pro-BDNF level between STZ + MS-275 [F (4,10) = 17.22, *P* > 0.999], and STZ + ROSI [F (4,10) = 17.22, *P* = 0.924, Figure 6a, b, c) in comparison with STZ + ROSI + MS-275.

 Meanwhile, there was a significant increase in m-BDNF level in STZ + ROSI + MS-275 [F (4,10) = 11.86, *P* = 0.01], STZ + MS-275 [F (4,10) = 11.86, *P* = 0.029], and STZ + ROSI [F (4,10) = 11.86, *P* = 0.049] compared with the STZ group, though the co-treatment group had a higher level compared with those means of monotherapy (Figure 6b).

 Moreover, a significant increase was seen in the m-BDNF to pro-BDNF ratio of STZ + ROSI + MS-275 [F (4,10) = 36.10], STZ + MS-275 [F (4,10) = 36.10], and STZ + ROSI [F (4,10) = 36.10], *P* < 0.001, compared with the STZ group (Figure 6c).

## Discussion

 The present study provides evidence of spatial and aversive memory impairment, reduction in m-BDNF/pro-BDNF, and elevation in M1 inflammatory microglia in the STZ-induced sporadic model of AD. Chronic co-administration of Rosiglitazone and MS-275 improved cognitive performance, promoted a shift in microglial polarization from the M1 to the M2 phenotype, and increased m-BDNF/pro-BDNF, however it was not significant compared with monotherapy.

 Previous studies have demonstrated that memory impairment induced by STZ is related to brain insulin resistance,^27^ amyloid deposition, oxidative stress, and elevated levels of inflammatory cytokines.^20^

 In the present study, we showed an increase in the pro-inflammatory M1 microglial phenotype 21 days after STZ administration, which accompanied by elevated pro-BDNF levels and cognitive dysfunction in this group. These findings are consistent with earlier reports showing that M1 microglia secrete pro-inflammatory mediators, including tumor necrosis factor-α (TNF-α), interleukin-12 (IL-12), interleukin-1β (IL-1β), and inducible nitric oxide synthase (iNOS), as well as contribute to amyloid-β (Aβ) plaque accumulation.^5^ These inflammatory processes have been implicated in synaptic loss and subsequent cognitive impairment.^28,29^

 To our knowledge, this is the first study demonstrating enhanced spatial and aversive memories following combined HDACi and PPARγ agonist treatment in an STZ-induced model of AD. These cognitive improvements were accompanied by an increase in M2 microglial polarization and elevated levels of mature BDNF in the co-treatment group, confirming an additive effect of the combined therapy. Therefore, the co-administration of MS275 and ROSI successfully improved both learning and memory, probably via activation of the anti-inflammatory M2 profile phenotype,^30^ and elevated levels of m-BDNF, which contributes to neuronal plasticity and memory formation.^18^ HDAC inhibition has been reported to repress the expression of AD-related genes, including NFκB, p53, tau,^31^ pro-BDNF, and activity-regulated cytoskeleton-associated protein (ARC).^32^ In addition, ROSI may exert neuroprotective effects through JAK1,3/STAT6,^33^ by downregulating neuroinflammation and oxidative stress,^34^ inhibiting NF-kB and iNOS, promoting neurogenesis,^35^ and facilitating amyloid-β (Aβ) clearance via phagocytosis.^36^

 On the other hand, elevation in m-BDNF following both co-treatment and single usage of MS275 and ROSI was associated with improvements in working and reference memories in the treated groups. Given the established role of BDNF in neural plasticity, synaptogenesis, neuroprotection,^37^ and metabolism,^38^ these findings suggest that mBDNF may act as a key mediator of the observed cognitive benefits. However, this study has some limitations that should be noted. First, our flow cytometry analysis was performed using two markers, so the results should be interpreted with caution. Second, we did not measure all downstream signaling of BDNF, which remain to be elucidated in future studies.

## Conclusion

 The results of this study indicate that co-treatment with ROSI and MS-275, at the early stage of AD, may represent a promising experimental strategy for investigating epigenetic-mediated neuroplasticity underlying memory. Moreover, this combined strategy may help alleviate cognitive deficits, at least partly by promoting a shift of microglia toward the anti-inflammatory M2 phenotype and increasing neurotrophic BDNF expression.

## Competing Interests

 The authors declare no conflict of interest.

## Ethical Approval

 The research protocol was approved by the Ethical Committee of Guilan University of Medical Sciences.
